# Multiple-Joint Pedestrian Tracking Using Periodic Models

**DOI:** 10.3390/s20236917

**Published:** 2020-12-03

**Authors:** Marzieh Dolatabadi, Jos Elfring, René van de Molengraft

**Affiliations:** Control Systems Technology Group, Department of Mechanical Engineering, University of Eindhoven, 5600 MB Eindhoven, The Netherlands; j.elfring@tue.nl (J.E.); m.j.g.v.d.molengraft@tue.nl (R.v.d.M.)

**Keywords:** tracking algorithm, pedestrian–car interaction, harmonic motion, kinematics estimation, joint tracking

## Abstract

Estimating accurate positions of multiple pedestrians is a critical task in robotics and autonomous cars. We propose a tracker based on typical human motion patterns to track multiple pedestrians. This paper assumes that the legs’ reflection and extension angles are approximately changing periodically during human motion. A Fourier series is fitted in order to describe the moving, such as describing the position and velocity of the hip, knee, and ankle. Our tracker receives the position of the ankle, knee, and hip as measurements. As a proof of concept, we compare our tracker with state-of-the-art methods. The proposed models have been validated by experimental data, the Human Gait Database (HuGaDB), and the Karlsruhe Institute of Technology and Toyota Technological Institute (KITTI) tracking benchmark. The results indicate that our tracker is able to estimate the reflection and extension angles with a precision of 90.97%. Moreover, the comparison shows that the tracking precision increases up to 1.3% with the proposed tracker when compared to a constant velocity based tracker.

## 1. Introduction

Pedestrian deaths account for more than one-fifth of road traffic deaths around the world [1]. Therefore, transportation systems, including vehicles and infrastructures, use various approaches to track pedestrians, due to the high number of fatalities. The tracking here is defined as estimating a pedestrian’s position and velocity. A tracker helps cars to plan their driving path and navigate safely. For example, suppose that a pedestrian is walking and does not notice a car near him/her. A tracker estimates the position and the velocity of the pedestrian. Subsequently, based on the tracker’s output, the car can alert the pedestrian or change its speed or path.

Therefore, tracking pedestrians is one of the critical tasks in robotics, non-autonomous, and autonomous cars. A tracker faces challenges, such as occlusion, noisy measurements, and a limited field of view. Moreover, tracking is not a trivial task when a tracker faces multiple pedestrians. Although some research has focused on this topic [2,3,4], tracking multiple pedestrians is still a challenge in urban areas [5].

A tracker must estimate the position and velocity of a pedestrian. To do this, trackers utilize a measurement model and a process model. A measurement model describes the relation between the pedestrian position and velocity that are estimated by the tracker and joint position measurements that are received from sensors. A process model describes how the pedestrian position and velocity are assumed to change over time. Earlier trackers [6,7,8,9] use the linear process or measurement models. The measurement model and process model are usually nonlinear in nonideal situations due to the occlusion, noisy measurements, and human moving patterns. Therefore, linear models should make assumptions, such as the routes are linear, pedestrians have linear movements, or pedestrians have movements with simple variations of direction [10]. These assumptions have negative consequences on tracking, and there is a probability that trackers with linear measurement models or process models are prone to fail during the tracking. Therefore, they are not always sufficient for tracking multiple pedestrians in an urban area [11]. The current state-of-the-art algorithms that track multiple pedestrians can be roughly divided into combined detection-tracking algorithms and tracking-by-detection paradigms.

In the combined detection-tracking algorithm, the typical approach in the literature is to use deep learning algorithms in order to track pedestrians while detecting them [12,13,14]. Although these kinds of trackers can match pedestrians anywhere in their sensors’ field of view, they likely produce more false positives [5]. Moreover, as a result of pedestrians’ nonlinear kinematic, this approach requires large datasets in practice. Because training on smaller datasets might lead to inaccurate tracks [15].

In the tracking-by-detection paradigm, there is an assumption that the detections are provided independently of a tracker. It means that the tracking-by-detection paradigm can draw a sharp distinction between the detection and tracking of pedestrians. Therefore, trackers of this paradigm can work with any detector. In this paradigm, after receiving detections, most of the trackers first define a bounding box (BB) around the pedestrian and localize the BB in a frame. The tracker associates the center of BB to pedestrians who were previously tracked [14]. As a result of the association, they can identify new pedestrians [16]. It means that detections that cannot be associated with tracked pedestrians can represent false detections or newly appeared pedestrians.

However, tracking a single point in a pedestrian’s body may produce more false-positives than a multiple point tracker due to noisy measurement and occlusion [17]. Tracking multiple joints of a body can offer a more attractive alternative than tracking a single position of each pedestrian. Suppose that a tracker receives several joints in a BB that overlap with each other. Subsequently, one pedestrian is tracked and the other joints are considered as a new pedestrian or false positives. The pre-requisite for these approaches is the ability to detect multiple joints from the sensor data.

In this paper, our goal is to track multiple pedestrians surrounding a car, even when there are occlusions. Therefore, we propose a pedestrian tracker that tracks pedestrians while using multiple joints instead of a single point. Our tracker belongs to the tracking-by-detection paradigm and our main goal is improving the measurement model and process model of a pedestrian tracker. In our tracker, a camera will be used for detecting pedestrians since cameras are typically available in automated vehicles. Our tracker should satisfy the following requirements:

- require an algorithm to associate noisy measurements with the position and velocity of pedestrians;
- contain models to predict and describe the movements of each pedestrian;
- the tracker should use images that it receives from a camera; and,
- the tracker should estimate the position of a pedestrian at a joint level.

Our tracker comprises a process model and a measurement model. The process model defines how our state vector is expected to change over time. Our measurement model describes how to make a connection between the state vector and detected joints. For each pedestrian, the measurement vector is the positions of joints in pixel coordinates. The contributions of this work are as follows:

- we propose a pedestrian tracker that can track multiple joints of pedestrians. We consider human kinematic constraints and a physical model to make a relation between joints. In our process model, we utilize time-varying Fourier series approximations and constant velocity assumptions;
- the state vector includes the position, the hip velocity of pedestrians, reflection, and extension angles between hip-knee and knee-ankle of each leg, and a pedestrian’s step frequency; and,
- we validate our tracker’s performance by evaluating it on experimental data, one gait dataset, and one tracking benchmark.

The rest of this paper is arranged, as follows: Section 2 discusses related work. In Section 3, we describe the general framework of our tracker. Section 4 introduces our proposed models. Section 5 describes how the issue of data association is handled. Section 6 contains the evaluation procedure, and, in Section 7, we validate our tracker. Section 8 presents conclusions and outlines future directions in this research.

## 2. Related Work

The first group of related works represents pedestrians by a single point. In order to track pedestrians [5], tracks a single point in the center of the body. In [18], based on the detection, the authors define the BB around each pedestrian in each image. Subsequently, they track the center of the BBs and estimate the position and velocity of the pedestrian. In [19], the researchers track each pedestrian as a point. In [20], the authors address the problem of detecting and tracking groups of people in RGB-D data. They consider each group to be a point. Therefore, they do not track each person individually.

Pedestrians can continuously change their position and direction. Therefore, the position of the BB varies with time. In a crowded area, there is a probability that a single point is occluding another point during tracking. Therefore, a tracker cannot receive any measurement regarding the occluded point [21]. Having more details about the detected pedestrian can decrease the false tracks. Pedestrians can be represented by more complicated models, including multiple joints, as an alternative to single-point trackers.

In [22], they develop a human skeleton tracking system. They use a constant velocity KF in order to track the positions of body joints [23] computes the displacements of 15 joints in the body relative to each other. They define the position of people based on the displacements of their joints [24] proposes a real-time method for tracking a pedestrian’s entire body and motion using unlabeled marker measurements. They track each joint based on the sensor attached to the body. To track, they use their measurement in a Kalman filter (KF). All of these trackers use a linear process model and motion model to track a human skeleton. A linear tracker can be used in a static camera [25]. Moreover, the linear models may produce more errors in their estimations when compared to the nonlinear model [26].

Among the various studies that track the pedestrian’s entire body, researchers have tracked them based on specific parts of the body [27] develops an eight joint skeleton model in order to track a person in a given video. They track each joint individually while using a KF. They assume that all joints move independently. Therefore, they define no relation between joints. One side effect of this assumption is that there is a probability that they use the joints of other pedestrians during occlusion. In [28], the authors propose a system for tracking both feet of pedestrians as they walked, based on multiple single-row laser scanners. In a crowded area, it is challenging for a detector to detect the feet. Therefore, a tracker requires more information regarding a pedestrian.

Several researchers have assessed the kinematic coupling between the hip and knee and ankle of a person walking in recent years [29,30,31]. Ref. [29] models the pedestrian leg as a pendulum with an EKF in order to estimate the displacement of a pedestrian. They attach two sensors on the right leg of a pedestrian to extract accelerations. Ref. [30] considers the periodic nature of walking, and they modify a bio-mechanical model with a first-order Taylor series expansion. Their state vector contains the angular position of the trunk relative to the vertical axis in a 2D plane, the angular position of the ankle relative to the hip joint, linear acceleration of the hip and the ankle. Using an IMU that was attached to the ankle joint, they measure the acceleration of the ankle and the angular position. Based on the measurements that were received from sensors, they calculate the coefficients of the Taylor series. Their process model has constant coefficients, whereas the coefficients should be varied based on age, weight, height, and gender. In [31], they use an accelerometer to measure movement angle, velocity, acceleration, and displacement of knees.

**Figure 1:**
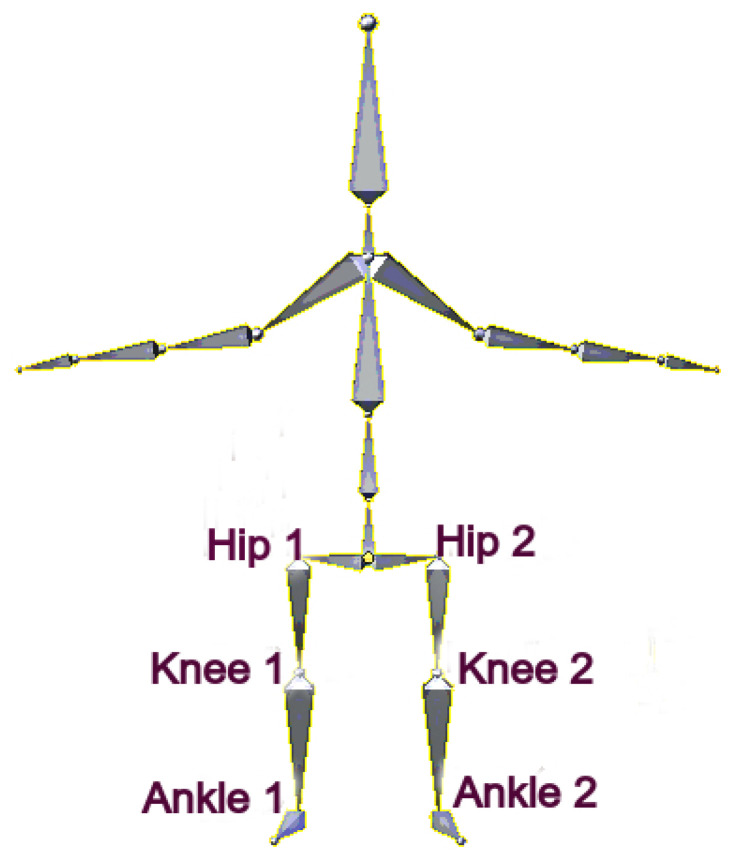
The joints of interest to detect and use in our tracker are at the ankles, knees, and hips.

This paper proposes a tracker that can be used for each age, weight, and gender. We track pedestrians’ legs instead of the whole body. We do this because of the simplicity of the shape of the legs. Legs form a large segment of a body. Hence, it is detectable, even from a low-resolution camera [32]. Moreover, we focus on tracking the position of six joints of pedestrians as they walk. The joints that will be used throughout this work are at the ankle, knee, and hip. Figure 1 shows those body joints.

To track, we use the Fourier series and EKF in our process model. With the Fourier series approximation, we compute the angles between each of the detected joints. On our measurement model, we utilize a two-link pendulum in order to make a relation between the joints. We use a state vector that facilitates using our process model and measurement model. At the same time, the state vector can be updated while using the measurements that are just explained. More details will be given in the following sections.

## 3. Pedestrian Tracker

**Figure 2:**
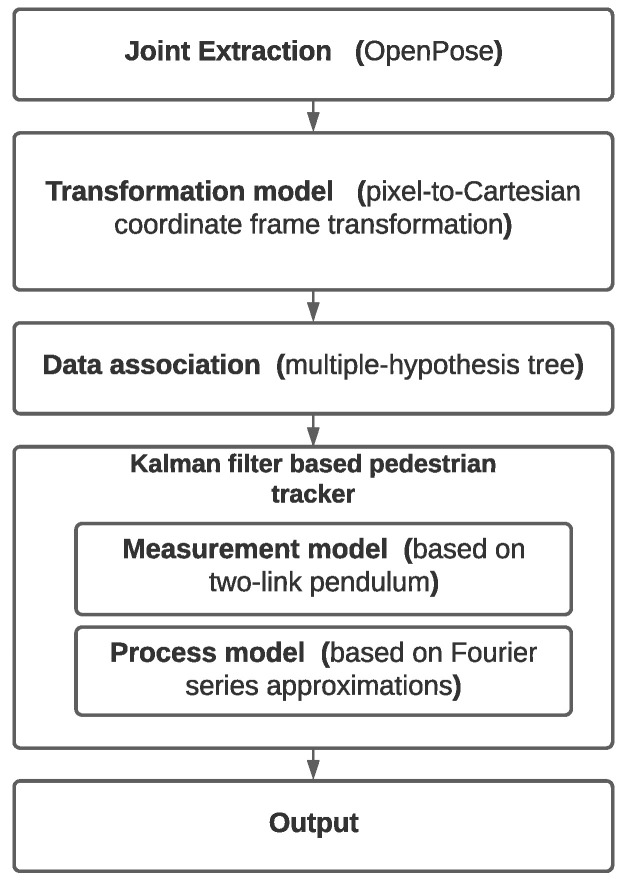
General framework of a tracker.

In this section, we introduce our pedestrian tracker. Figure 2 shows its conceptual composition. The joint measurements is input to our pedestrian tracker. There are libraries to extract joints [33,34,35,36]; one of the most popular ones is OpenPose [33]. In this work, OpenPose is utilized in order to detect the joints. OpenPose provides a position vector for each joint in pixel coordinates.

In our work, after receiving the data about the joints, we implement a pixel-to-Cartesian coordinate frame transformation. To perform this transformation, we require knowledge of the camera’s orientation with respect to a pedestrian’s joints. Each joint has a frame with an x parallel to the ground and y pointing upwards. Moreover, we need the camera’s focal length and each joint position in the pixel coordinates. Based on the Dutch population, we assume an average height of 177 cm for pedestrians. Having no depth information regarding a pedestrian was the only reason to make this assumption. Afterwards, we solve a backward perspective projection model equation [37]. We compute the length of a pedestrian leg when we receive the positions of a pedestrian’s joints in the Cartesian coordinate frame for the first time. Subsequently, we assume that this length is constant and equal for the two legs of a pedestrian.

In the data association block, we use a multiple-hypothesis tree, as implemented in [38], to match each leg of a detected pedestrian with pedestrians that our tracker is already tracking. We used an EKF in order to track and predict the position of the joints of pedestrians based on detections of individual joints and nonlinear models. The EKF comprises a measurement and a process model.

**Figure 3:**
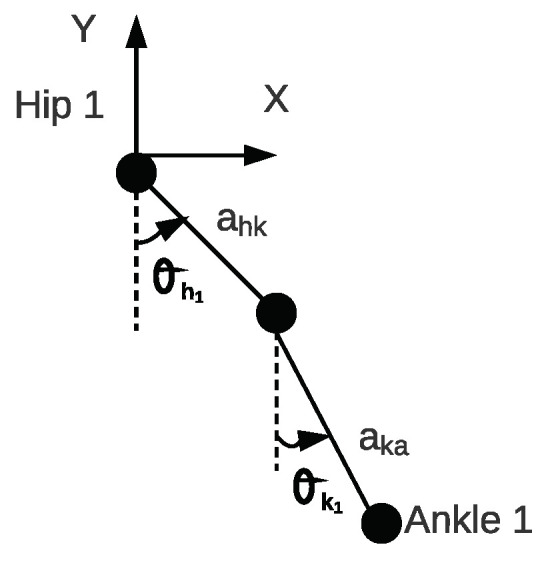
The right leg from the side view in a schematic way. $a_{hk}$ corresponds to a length between the hip and the knee. $a_{ka}$ is a length between the knee and ankle. Both of the angles are defined as positive in counterclockwise direction.

As mentioned, our tracker should track a pedestrian, even if our detector does not detect a joint. For example, it should estimate the ankle’s position based on the hip where a detector cannot detect the ankle, but it detects the hip. To meet the requirement, we use a two-link pendulum to define each joint’s position with respect to the other joints. To do this, we require angles between joints. $\theta_{H_{1}}$ and $\theta_{K_{1}}$, represent the hip and knee flexion and extension angles in the right leg. Figure 3 shows these angles. The angles $\theta_{H_{2}}$ and $\theta_{K_{2}}$ have the same definition in the left leg.

Based on the output of the blocks, our tracker delivers each pedestrian’s hip, knee, and ankle position, the velocity of the hip with respect to the camera, the angles of joints, and the step frequency of the pedestrian.

## 4. Proposed Models

In our process model, we use the periodic nature of walking and the constant velocity model to describe how the state changes over time. We assume that, during walking, the hip, knee, and ankle lie on a 2D plane. In our measurement model, we exploit the relations shown in Figure 3.

### 4.1. Process Model

To define the process model, our assumptions are as follows:

1. In gait analysis, walking is assumed to be periodic [39].
2. In between two frames, we assume that the frequency of the angles is constant.
3. There is a linear relation between walking velocity and frequency.
4. Both of the legs move with the same frequency during one continuous walking.
5. The hip velocity of a pedestrian in the Y direction is zero.
6. The two joints of the hip have the same linear velocity in the X direction.
7. In each leg, the frequency of the angles is equal. It means that the rate of completing a stride is equal in the joints of a leg.

Based on our assumptions, each angle could be modeled as a periodic signal. The Fourier series can approximate such a periodic function as a function of time. Hence, it is possible to use a Fourier series to propagate each angle [40]. Additionally, based on our assumptions, we use a frequency-velocity model to estimate the motion of pedestrians. The process model has been structured to be represented while using the following equation:

(1) x(t)=f(x(t−1))+w(t),w(t)∼N(0,Q)

where *x* is a state vector, *f* is a non-linear state transition function that computes the predicted state from the previous estimate, and *w* is process noise. We assume that it is zero-mean white noise with a known covariance matrix. *Q* is the covariance matrix and it is constant, because the upper value of *Q* can obtain an acceptable estimating precision [41].The state vector for each pedestrian is defined as:

$$\begin{array}{c} x\left(t\right)=[X_{h_{1}}\left(t\right),Y_{h_{1}}\left(t\right),V_{x_{h}}\left(t\right),SF\left(t\right),\theta_{H_{1}}\left(t\right),\omega_{H_{1}}\left(t\right),\theta_{K_{1}}\left(t\right),\\ \omega_{K_{1}}\left(t\right),X_{h_{2}}\left(t\right),Y_{h_{2}}\left(t\right),\theta_{H_{2}}\left(t\right),\omega_{H_{2}}\left(t\right),\theta_{K_{2}}\left(t\right),\omega_{K_{2}}{\left(t\right)]}^{T} \end{array}$$

where:

- $X_{h_{1}}$ and $Y_{h_{1}}$ are the hip position of the right leg in two directions at time *t* with respect to the measurement sensor.
- $V_{x_{h}}\left(t\right)$ is the linear velocity of the hip at time *t*.
- SF(t) is the frequency of the joints at time *t*.
- $\omega_{H_{1}}\left(t\right)$ is a time derivative of $\theta_{H_{1}}\left(t\right)$ and $\omega_{K_{1}}\left(t\right)$ is a time derivative of $\theta_{K_{1}}\left(t\right)$ in the right leg.
- $X_{h_{2}}$ and $Y_{h_{2}}$ are the hip position of the left leg in two directions at time *t* with respect to the measurement sensor.
- $\omega_{H_{2}}\left(t\right)$ is a time derivative of $\theta_{H_{2}}\left(t\right)$ and $\omega_{K_{2}}\left(t\right)$ is a time derivative of $\theta_{K_{2}}\left(t\right)$ in the left leg.

Based on our third assumption, we can use a linear model in order to propagate the velocity of the hip joints. Based on [42], a first-order Fourier series can cover hip, knee, and ankle position with an accuracy of 96%, 93%, and 89%. Therefore, we utilize the first order of the Fourier series. It means that the maximum amplitude of angles and the initial phase angles are constant. The non-linear state transition function for each state can be defined, as follows:

(2) $$\begin{array}{cc} X_{h_{1}}\left(t+1\right) & =V_{x_{h}}\left(t\right)dt+X_{h_{1}}\left(t\right)\\ Y_{h_{1}}\left(t+1\right) & =Y_{h_{1}}\left(t\right)\\ V_{x_{h}}\left(t+1\right) & =V_{x_{h}}\left(t\right)\\ SF(t+1) & =SF(t)\\ \theta_{H_{1}}\left(t+1\right) & =A_{H_{1}}sin\left(SF\left(t+1\right)\left(t+1\right)+\phi_{H_{1}}\right)\\ \omega_{H_{1}}\left(t+1\right) & =A_{H_{1}}SF\left(t+1\right)cos\left(SF\left(t+1\right)\left(t+1\right)+\phi_{H_{1}}\right)\\ \theta_{K_{1}}\left(t+1\right) & =A_{K_{1}}sin\left(SF\left(t+1\right)\left(t+1\right)+\phi_{K_{1}}\right)\\ \omega_{K_{1}}\left(t+1\right) & =A_{K_{1}}SF\left(t+1\right)cos\left(SF\left(t+1\right)\left(t+1\right)+\phi_{K_{1}}\right)\\ X_{h_{2}}\left(t+1\right) & =V_{x_{h}}\left(t\right)dt+X_{h_{2}}\left(t\right)\\ Y_{h_{2}}\left(t+1\right) & =Y_{h_{2}}\left(t\right)\\ \theta_{H_{2}}\left(t+1\right) & =A_{H_{2}}sin\left(SF\left(t+1\right)\left(t+1\right)+\phi_{H_{2}}\right)\\ \omega_{H_{2}}\left(t+1\right) & =A_{H_{2}}SF\left(t+1\right)cos\left(SF\left(t+1\right)\left(t+1\right)+\phi_{H_{2}}\right)\\ \theta_{K_{2}}\left(t+1\right) & =A_{K_{2}}sin\left(SF\left(t+1\right)+\phi_{K_{2}}\right)\\ \omega_{K_{2}}\left(t+1\right) & =A_{K_{2}}SF\left(t+1\right)cos\left(SF\left(t+1\right)\left(t+1\right)+\phi_{K_{2}}\right) \end{array}$$

where dt is a time difference between discrete time steps *t* and (t+1). $\phi_{H_{1}}$, $\phi_{K_{1}}$, $\phi_{H_{2}}$, and $\phi_{K_{2}}$ are the initial phase angles of hip and knee in both legs. $A_{H_{1}}$, $A_{K_{1}}$, $A_{H_{2}}$, and $A_{K_{2}}$ are the maximum amplitude of angles in both legs. The maximum amplitudes and the initial phase angles are different in males and females [43]. Therefore, we estimate the angles and their rate independence of them. To do it, first, we use the cosine and sine expansion. According to the constant frequency assumption and the expansions, we rewrite the $\omega_{H_{1}}$ and $\theta_{H_{1}}$ from (Equation (2)), as follows:

(3) $$\begin{array}{cc} \; & \theta_{H_{1}}\left(t+1\right)=A_{H_{1}}[sin\left(SF\left(t\right)t+\phi_{H_{1}}\right)cos\left(SF\left(t\right)\right)\\ & +cos\left(SF\left(t\right)t+\phi_{H_{1}}\right)sin\left(SF\left(t\right)\right)]\\ & =A_{H_{1}}\left(C1.C2\right)+A_{H_{1}}\left(C3.C4\right) \end{array}$$

$$\begin{array}{cc} \; & C1=sin(SF\left(t\right)t+\phi_{H_{1}})\\ \; & C2=cos(SF(t))\\ \; & C3=cos(SF\left(t\right)t+\phi_{H_{1}})\\ \; & C4=sin(SF(t)) \end{array}$$

As can been seen, we can make a relation between C1 and $\theta_{H_{1}}\left(t\right)$ and between C3 and $\omega_{H_{1}}\left(t\right)$. Therefore, we have:

(4) $$\theta_{H_{1}}\left(t+1\right)=\theta_{H_{1}}\left(t\right)C2+\frac{\omega_{H_{1}}\left(t\right)}{SF(t)}C4$$

Similar to (4), we rewrite the $\omega_{H_{1}}\left(t+1\right)$, as:

(5) $$\omega_{H_{1}}\left(t+1\right)=\frac{d\theta_{H_{1}}}{dt}=\omega_{H_{1}}\left(t\right)tC2-\theta_{H_{1}}\left(t\right)SF\left(t\right)C4$$

We repeat (Equations (4) and (5)) for the right knee and for the left leg.

### 4.2. Measurement Model

The measurement model has been structured to be represented using the following equation:

(6) z(t)=h(x(t))+v(t),v(t)∼N(0,R)

*h* is used to compute the predicted measurement position from the predicted state. *v* is measurement noise. We assume that it is zero-mean white noise with a known covariance matrix. *R* is the covariance matrix of measurements.

The structure of the human lower limb acts as a kinetic chain during walking. Therefore, the position of the hip joint interacts with the knee and ankle position. We used homogeneous transformation matrices to transform the position of knee and ankle joints to the hip joint. The matrices are computed, as follows:

(7) $$T_{K}^{H}=\left[\begin{array}{ccc} cos(\theta_{H_{1}}\left(t\right)) & -sin(\theta_{H_{1}}\left(t\right)) & a_{hk}sin\left(\theta_{H_{1}}\left(t\right)\right)\\ sin(\theta_{H_{1}}\left(t\right)) & cos(\theta_{H_{1}}\left(t\right)) & -a_{hk}cos\left(\theta_{H_{1}}\left(t\right)\right)\\ 0 & 0 & 1 \end{array}\right]\;T_{A}^{K}=\left[\begin{array}{ccc} cos(\theta_{K_{1}}\left(t\right)) & -sin(\theta_{K_{1}}\left(t\right)) & a_{ka}sin\left(\theta_{K_{1}}\left(t\right)\right)\\ sin(\theta_{K_{1}}\left(t\right)) & cos(\theta_{K_{1}}\left(t\right)) & -a_{ka}cos\left(\theta_{K_{1}}\left(t\right)\right)\\ 0 & 0 & 1 \end{array}\right]$$

where $T_{K}^{H}$ is a transformation matrix of the knee position to the hip joint, and $T_{A}^{K}$ transforms the ankle joint to the knee joint. $a_{hk}$ corresponds to a length between the hip and the knee. $a_{ka}$ is a length between the knee and ankle. We assume these two lengths are equal for two legs. $T_{A}^{H}$ is a transformation of the ankle joint to the hip joint. $T_{A}^{H}$ is computed by multiplying $T_{K}^{H}$ and $T_{A}^{K}$.

Figure 3 illustrates the right leg from the side view in a schematic way. We repeat the same matrices for the left leg and, then, based on the transformation matrices, the following equations are extracted, which are the joints’ positions with respect to the camera frame.

(8) $$\begin{array}{cc} X_{h_{1}}\left(t\right) & =X_{h_{1}}\left(t\right)+a_{hk}sin\left(\theta_{H_{1}}\left(t\right)\right)\\ Y_{h_{1}}\left(t\right) & =Y_{h_{1}}\left(t\right)-a_{hk}cos\left(\theta_{H_{1}}\left(t\right)\right)\\ X_{a_{1}}\left(t\right) & =X_{h_{1}}\left(t\right)+a_{hk}sin\left(\theta_{H_{1}}\left(t\right)\right)\\ & +a_{ka}sin\left(\theta_{H_{1}}\left(t\right)+\theta_{K_{1}}\left(t\right)\right)\\ Y_{a_{1}}\left(t\right) & =Y_{h_{1}}\left(t\right)-a_{hk}cos\left(\theta_{H_{1}}\left(t\right)\right)\\ & -a_{ka}cos\left(\theta_{H_{1}}\left(t\right)+\theta_{K_{1}}\left(t\right)\right)\\ X_{k_{2}}\left(t\right) & =X_{h_{2}}\left(t\right)+a_{hk}sin\left(\theta_{H_{2}}\left(t\right)\right)\\ Y_{k_{2}}\left(t\right) & =Y_{h_{2}}\left(t\right)-a_{hk}cos\left(\theta_{H_{2}}\left(t\right)\right)\\ X_{a_{2}}\left(t\right) & =Y_{h_{2}}\left(t\right)+a_{hk}sin\left(\theta_{H_{2}}\left(t\right)\right)\\ & +a_{ka}sin\left(\theta_{L_{1}}\left(t\right)+\theta_{L_{2}}\left(t\right)\right)\\ Y_{a_{2}}\left(t\right) & =Y_{h_{2}}\left(t\right)-a_{hk}cos\left(\theta_{H_{2}}\left(t\right)\right)\\ & -a_{ka}cos\left(\theta_{H_{1}}\left(t\right)+\theta_{K_{2}}\left(t\right)\right)\\ h(x(t)) & =[X_{h_{1}},Y_{h_{1}},X_{k_{1}},Y_{k_{1}},X_{a_{1}},Y_{a_{1}},\\ & X_{h_{2}},Y_{h_{2}},X_{h_{2}},Y_{h_{2}},X_{h_{2}},Y_{h_{1}}{]}^{T} \end{array}$$

where $X_{k_{1}}$ and $Y_{k_{1}}$ are the knee positions and $X_{a_{1}}$ and $Y_{a_{1}}$ are the ankle position of the right leg, which are computed using the hip positions and a double pendulum model. $X_{h_{2}}$, $Y_{h_{2}}$, $X_{h_{2}}$, and $Y_{h_{2}}$ have the same definition for the left leg. *z* is our measurement vector. We can use a linear model to track the hip because the hip’s angular displacement is insignificant. In contrast, the knee and ankle have angular displacement; therefore, we utilize angles to compute other joints’ positions. For other joints, we consider the effect of nonlinear motion.

## 5. Data Association

In this section, we describe how the tracker solves the association problem while using a multiple-hypothesis tree (MHT) [44]. Data association is the process of matching newly detected pedestrians with pedestrians that were already being tracked. Moreover, data association determines which of the detected legs is the right leg and which one is the left leg. To associate data, MHT generates a hypothesis tree with several branches.

Each measurement can be associated with an existing pedestrian, clutter, or a pedestrian that was not tracked before. Therefore, each branch is a collection with hypotheses. For each measurement, each branch can be formed with different possible associations. Every hypothesis contains a list of pedestrians and the estimation of their state vector. Hypotheses are considered in parallel. Therefore, data association decisions can be deferred until uncertainties on data association are resolved. The tree expands by receiving a new measurement at a time of t+1. The probability of each hypothesis is computed in order to pick the most probable hypothesis and keep the tree size bound.

## 6. Performance

We utilize Multiple Object Tracking Precision (MOTP) to have a clear and understandable evaluation [36]. MOTP quantifies the tracker’s ability to determine a pedestrian’s exact position.

(9) $$MOTP=\frac{\sum_{i,t}d_{t}^{i}}{\sum_{t}c_{t}}$$

where

- $c_{t}$ is the total number of pedestrians; and,
- $d_{t}$ is the total position error for matched pedestrians.

**Figure 4:**
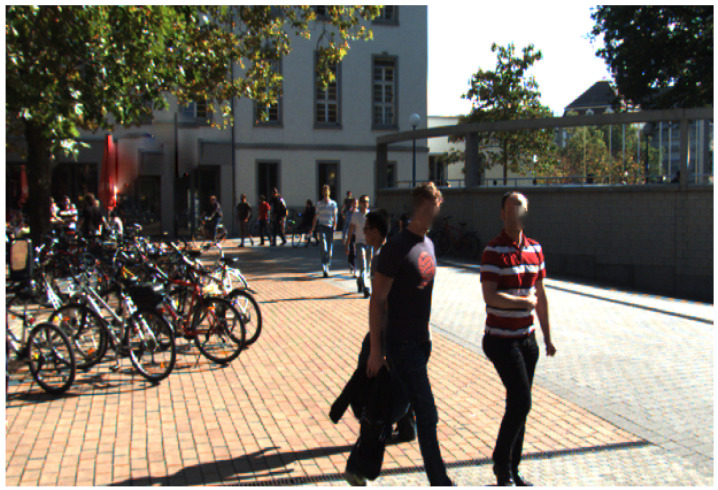
A campus pedestrians tracking.The training sequences number 17 of the Karlsruhe Institute of Technology and Toyota Technological Institute (KITTI) tracking benchmark.

**Figure 5:**
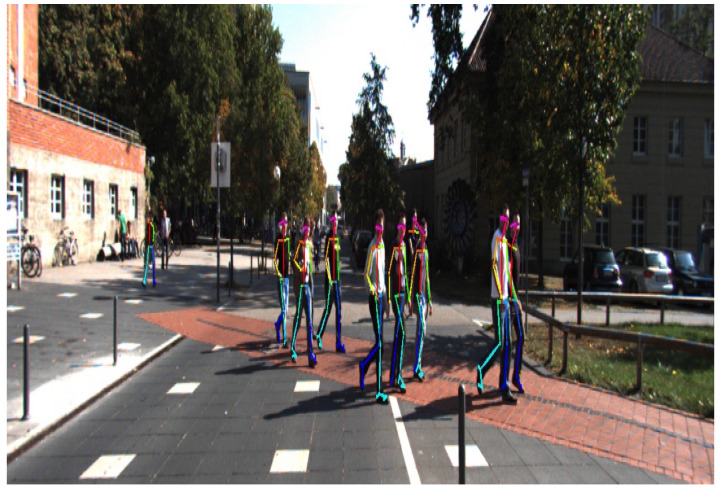
Result of OpenPose joints detection.The training sequences number 16 of the KITTI tracking benchmark.

To evaluate, we use the human gait dataset (HuGaDB) [45] and the Karlsruhe Institute of Technology and Toyota Technological Institute (KITT)I tracking benchmark [36]. HuGaDB collects data from a body sensor network of six wearable accelerometers that were located on the right and left legs. The KITTI benchmark consists of 21 training sequences and 29 test sequences. They collect data at 10 Hz with a camera mounted on a moving car in a city, residential area, campus, and road. Figure 4 shows one of the individual benchmarks of KITTI. An output of OpenPose in the KITTI benchmark is presented in Figure 5. OpenPose links all joints that belong to the person and assigns them different colors. Figure 5 shows these links.

## 7. Experimental Evaluation

In this section, we evaluate our tracker with the HuGaDB dataset, experimental data, and the KITTI tracking benchmark. In the first part, we prove that our tracker can determine an acceptable MOTP. We then compare our tracker with another tracker that is used in [22]. For both trackers, we implemented the same data association and measurements. In the last part, we compare our results with the state-of-the-art [5,14,19,36].

### 7.1. HuGaDB Dataset

In this part, our objective is validating the models that we implement in EKF. We compare the result of $\theta_{H_{1}}$, $\theta_{K_{1}}$, $\omega_{H_{1}}$, $\omega_{K_{1}}$ in our tracker and the HuGaDB dataset for validation.

In HuGaDB, they placed six inertial sensors and electromyography (EMG) sensors on the right and the left pedestrians’ thigh, shin, and foot. This dataset provides detailed gait data of the legs during walking and running [45]. The dataset contains the measurements that we need in our tracker. Therefore, we have the ground truth (GT) data for all the joints while using the HuGaDB dataset. GT is calculated from the acceleration of the sensors attached to the leg [45]. The sensors send their output to our tracker. Therefore, we do not use Openpose to detect the joints of a pedestrian. Hence, we can prove that the output of our state vector has a high MOTP.

**Figure 6:**
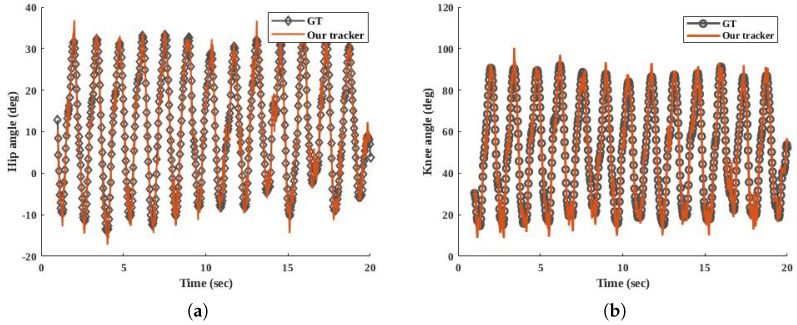
Measured and estimated results of our tracker for the right leg of one participant in HuGaDB dataset who was a 24-year old male with 177 cm stature and 75 kg body mass. (**a**) shows the angle between thigh and hip ($\theta_{H_{1}}$). (**b**) shows ($\theta_{K_{1}}$) measured using the accelerometers and estimated using our tracker.

Figure 6 draws comparisons for the right leg between the GT and our tracker. The ground truth angles come from two gyroscope sensors. The GT values of $\theta_{H_{1}}$ and $\theta_{K_{1}}$ were calculated as [31]. The results in the part (a) and (b) of Figure 6 show that this participant completes thirteen cycles during his walking in 20 s. Each maximum peak shows a swing phase of his right leg, and the minimum peaks indicate the stance phases.

**Figure 7:**
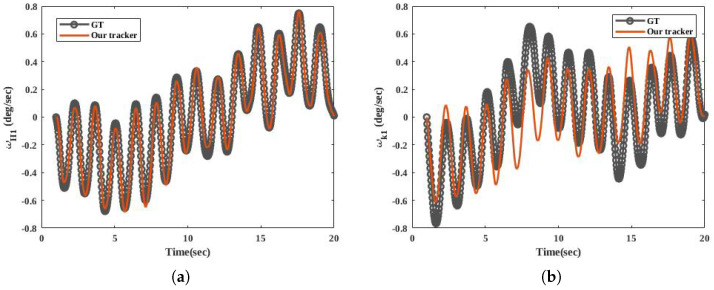
Measured and estimated results of our tracker for the right leg of one participant in HuGaDB dataset who was a 24-year old male with 177 cm stature and 75 kg body mass. (**a**) indicates $\omega_{H_{1}}$ and (**b**) compares the $\omega_{K_{1}}$ between the tracker and the dataset.

Based on Figure 6, our tracker estimates a consistent angle pattern. This consistent pattern means that the first order of the Fourier series can cover the walking pattern. Figure 7 provides a visual representation of $\omega_{H_{1}}$ and $\omega_{K_{1}}$ in our tracker and the dataset.

In Figure 7a,b, the zero values indicate no angular movement at that time, and the peaks occur during stance and swing phases.There are clear errors in Figure 7b. These errors may arise from facts, such as the sensor having a vibration, the attached sensors being mounted in slightly different positions, or the knee’s process model should be different. The probability of the third fact is low, since, in Figure 6b, we estimate the knee angle close to the GT. Unlike deep learning trackers, our tracker is explainable. It explains the variation of the state vector and the measurement, since we can demonstrate the models of our tracker.

Table 1 gives the MOTP of our tracker for both legs of all participants. The result of MOTP indicates that our tracker can compute the angles and their rate close to the ground truth data. Based on (9), MOTP is a function of the estimation total error. Therefore, for all of the participants, the mean error in angle is 3.6${}^{\circ }$.

### 7.2. Comparison

**Figure 8:**
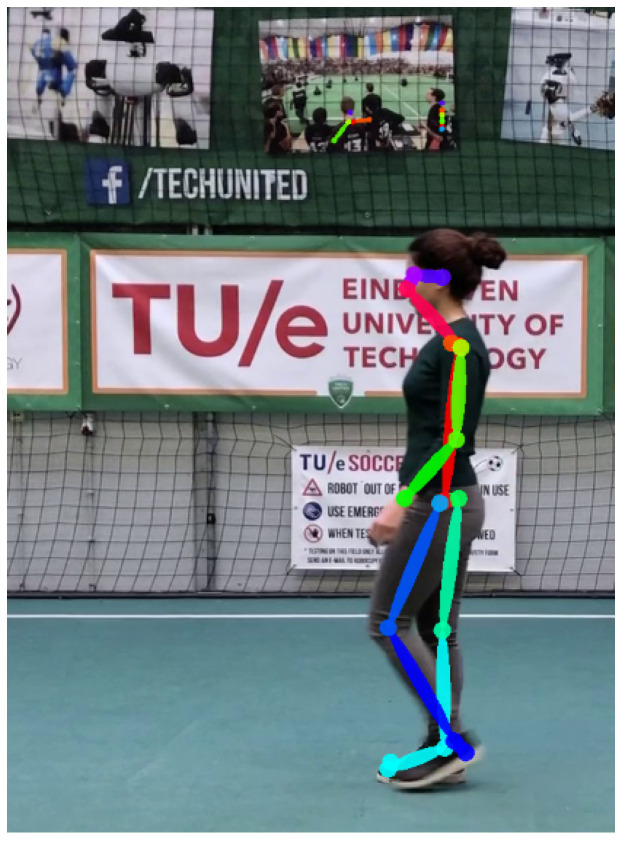
A pedestrian is crossing in front of a camera with constant velocity.

In order to compare the advantages of our process model and measurement model with another tracker, we replaced them with models that are used in [22]. It means that the data association part of the two trackers is the same. Then, we defined a test scenario. In this scenario, a person was crossing a line at a constant speed for a given time. Then proceeded a curve to turn back to the starting point. The camera was fixed during this test, and the camera’s distance to the joints and the crossing distance was known. Figure 8 shows an illustrative camera image with the detections that were used by both trackers.

**Figure 9:**
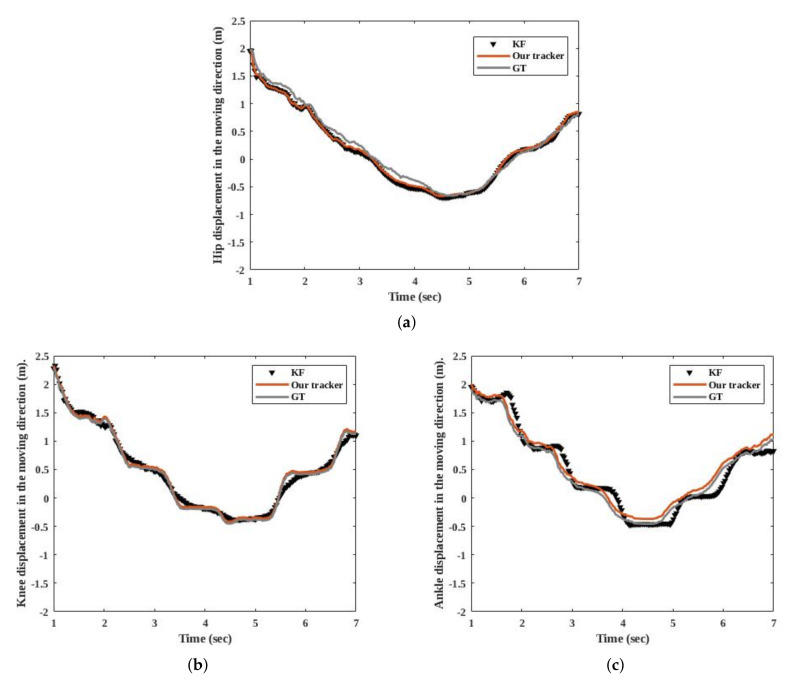
Measured and estimated results of the trackers for a person who was crossing in front of the camera. (**a**) shows the position of the hip joint in the left leg. (**b**) indicates the position of the knee joint in the left leg, and (**c**) compares the two trackers with each other based on the position of the ankle of the left leg.

In Ref. [22], they use a constant velocity KF; their measurement vector contains the positions of each joint. Figure 9 compares GT with the two trackers. Figure 9 shows that the joints move roughly with a constant velocity during the swing phase, they are constant during the stance phase, and then they move again.

Although [22] and our tracker used the same data association and measurements, there is a difference between GT and [22] during turning. This difference is due to the use of a linear measurement model and a process model in [22]. Similar to [22], we also use a constant velocity model to compute the hip position. For other joints, we consider the effect of nonlinear motion.

For a quantitative comparison, Table 2 gives MOTP. Table 2 proves that our tracker estimates the positions of the hip with more precisionm since it has a higher MOTP than the tracker used in [22]. We achieve a relatively high MOTP, because the pedestrian walks with both linear and nonlinear patterns. It indicates that our process model and measurement model help to improve pedestrian tracking.

### 7.3. KITTI Dataset

As GT data, this dataset provides the center of a BB around the pedestrian in an urban environment. We assume that the center of a BB is equal to the center of the body. In order to compare our tracker with state-of-the-art, we calculate the center of the pedestrian in relation to the hip joints. Therefore, we only compare the center with the GT data. Table 3 compares the results of our tracker to the state-of-the-art algorithms for pedestrian tracking of the KITTI benchmark. The results of the algorithms presented in Table 3 are published on the KITTI website.

There are multiple reasons for having a MOTP with a margin of 2.95%. The reasons are as follows:

- Lack of GT for the joints.
- OpenPose.

As mentioned before, we assumed that all of the pedestrians have the same height. This assumption can produce an error. For example, when the sensors detect a pedestrian with 160 cm height and the tracker assumes an average height of 177 cm, MOTP would be different from reality.

Moreover, we assumed that the center of a BB is in the center of the body. This assumption affects all properties, such as size, location, orientation, and even pose of a pedestrian. For example, when a pedestrian has no symmetry pose from a detector point of view, this assumption can produce an error. We revalidated our tracker with new heights in order to explore the effects of these assumptions on the tracker. Once, we assumed that the average height is 150 cm. Subsequently, the average height is 190 cm. We repeated the benchmark with these two new values and computed MOTP. The results proved that the MOTP of KITTI is different based on the height value. These two assumptions can change the MOTP of KITTI for 2.95%. The variation of 2.95% in Table 3 shows that the comparison is not entirely fair. Other methods did not require the depth information at a joint level. Therefore, they only used the center of BBs and did not require estimating the height.

**Figure 10:**
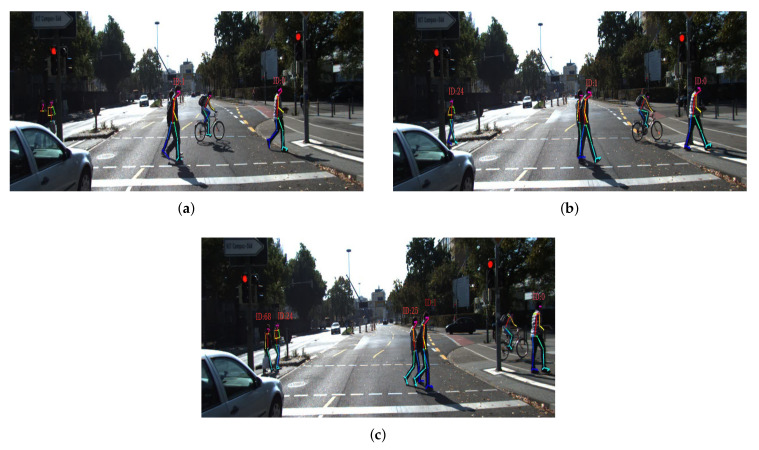
In (**a**), one pedestrian occludes another one, and the legs of two pedestrians are occluded by a car. In (**b**), one of the occluded pedestrians is in the field of view of the camera. In (**c**), OpenPose detects their joints. In situations such as (**a**) and (**b**), OpenPose can not detect pedestrians, affecting the results of Multiple Object Tracking Precision (MOTP).

Figure 10a,b show a situation that as result of occlusion OpenPose cannot detect the legs of pedestrians. After one frame, OpenPose detects all of them, as shown in Figure 10c. In these kinds of situations, that OpenPose does not perform well, our tracker can be negatively affected.

The pedestrian’s height assumption is strong. It can be mitigated while using the information from the stereo cameras or point cloud data. Therefore, we used Velodyne point data to decrease the height assumption’s effect. For one experiment in the testing part of KITTI, we matched the Velodyne point cloud data’s timestamps with the camera data. Therefore, we had the pedestrian’s distance to the car. Subsequently, we estimated our MOTP for that specific experiment. The result shows that we can increase the MOTP up to 0.72% for that experiment. Other methods in Table 3 compute MOTP without stereo cameras or point cloud data. Therefore, it is not fair that we compute MOTP while using these data.

**Figure 11:**
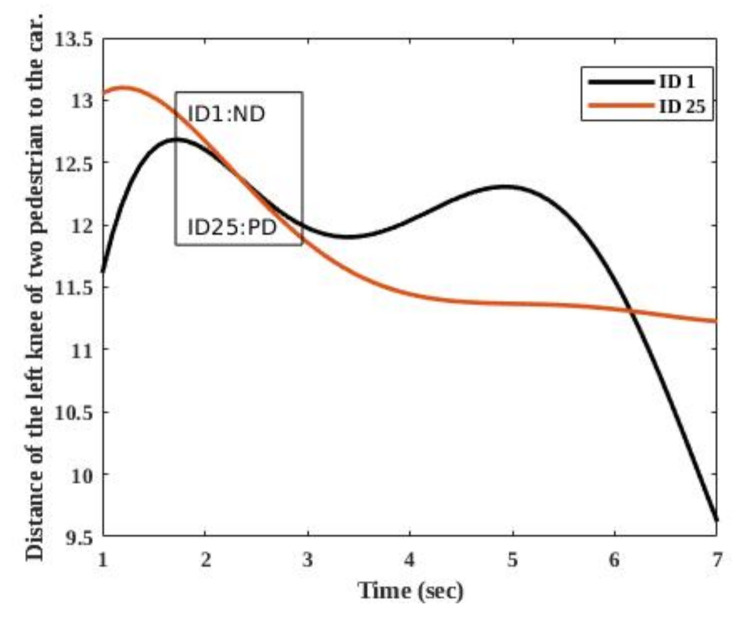
The result of the tracking two pedestrians in the sequences number 15 of the KITTI tracking benchmark. The vertical axes of the figure is the displacement of the pedestrians to a standing car. In the figure, ND means no detection and PD indicates partial detection.

However, KITTI does not provide the position at multiple joints levels. The benchmark was recorded in crowded areas, and pedestrians often occlude each other. Therefore, we use the dataset in order to show the performance of our tracker in challenging situations that are representative for the application domain. Figure 11 shows the tracking results of two pedestrians, pedestrian 1 and pedestrian 25, in Figure 10c. The vertical axis in Figure 11 is the distance of the two pedestrians’ left knees relative to the car, and the horizontal axis indicates time. The two pedestrians crossed the road in seven seconds. It should be noted that, as mentioned before, the goal of Figure 11 is indicating that our tracker can estimate a distance of a pedestrian continuously, even during occlusion. Therefore, the peaks in the figure do not mean swing or stance phases.

In Figure 11, there is a period that our tracker receives partial detection (PD) for the pedestrian with ID 1 and no detection (ND) for the pedestrian with ID 25. Our tracker estimates the distance of the knee to the standing car during PD and ND. Figure 11 shows that our tracker is able to track a pedestrian, even during an occlusion. We chose the left knees, because, based on Figure 10, the left sides of these two pedestrians are not always visible. Therefore, estimating the position of the left knee was more difficult.

## 8. Conclusions

We introduced a pedestrian tracker in order to track pedestrians’ position as a two-link pendulum with an Extended Kalman Filter. The tracker is an explainable tracker, it receives skeleton data of each pedestrian. Subsequently, based on the human anatomy, we model the relation between skeleton data. Our tracker can track six different joints of each pedestrian. Tracking with multiple joints helps the tracker to achieve more information regarding a pedestrian. Our evaluations show that this tracker can track pedestrians in urban areas during occlusion and turning.

In future work, we will extend the proposed method to support joints along the entire body, such that partial occlusions are expected to be handled even better. 

## Figures and Tables

**Table 1:** Evaluation metrics for tracking all 40 participates in HuGaDB dataset.

Measurement	MOTP
Angle	90.97%
Angular velocity	84.53%

**Table 2:** Evaluation metrics for a person tracking based on the sequence of images.

Method	MOTP
Our tracker	98.60%
KF [22]	97.37%

**Table 3:** Multiple Target tracking evaluation metrics for KITTI Pedestrian tracking benchmark.

Method	MOTP
Our tracker	74.03 ± 2.95%
SRK-ODESA [36]	75.07%
HWFD [36]	74%
Quasi-Dense [36]	73.99%
CenterTrack + MTFF [36]	75.02%
TuSimple [19]	71.93%
VVteam [14]	72.29%
MDP [36]	70.36%
